# Evaluation of the safety and efficacy of pregabalin in older patients with neuropathic pain: results from a pooled analysis of 11 clinical studies

**DOI:** 10.1186/1471-2296-11-85

**Published:** 2010-11-05

**Authors:** David Semel, T Kevin Murphy, Gergana Zlateva, Raymond Cheung, Birol Emir

**Affiliations:** 1 Pfizer Global Pharmaceuticals, New York, NY, USA

## Abstract

**Background:**

Older patients are typically underrepresented in clinical trials of medications for chronic pain. A post hoc analysis of multiple clinical studies of pregabalin in patients with painful diabetic peripheral neuropathy (DPN) or postherpetic neuralgia (PHN) was conducted to evaluate the efficacy and safety of pregabalin in older patients.

**Methods:**

Data from 11 double-blind, randomized, placebo-controlled clinical studies of pregabalin in patients with DPN or PHN were pooled. Efficacy outcomes included change in Daily Pain Rating Scale score, ≥30% and ≥50% responders, and endpoint pain score ≤3. Safety was based on adverse events (AEs). Primary efficacy was analyzed by analysis of covariance with terms for treatment, age category, protocol, baseline pain, and treatment-by-age category interaction.

**Results:**

2516 patients (white, n = 2344 [93.2%]; men, n = 1347 [53.5%]; PHN, n = 1003 [39.9%]; pregabalin, n = 1595) were included in the analysis. Patients were grouped by age: 18 to 64 years (n = 1236), 65 to 74 years (n = 766), and ≥75 years (n = 514). Baseline mean pain and sleep interference scores were comparable across treatment and age groups. Significant improvements in endpoint mean pain were observed for all pregabalin dosages versus placebo in all age groups (p ≤ 0.0009), except for the lowest dosage (150 mg/day) in the youngest age group. Clinically meaningful pain relief, defined as ≥30% and ≥50% pain response, was observed in all age groups. The most common AEs were dizziness, somnolence, peripheral edema, asthenia, dry mouth, weight gain, and infections. The relative risks for these AEs increased with pregabalin dose, but did not appear related to older age or type of neuropathic pain.

**Conclusions:**

Pregabalin (150-600 mg/day) significantly reduced pain in older patients (age ≥65 years) with neuropathic pain and improvements in pain were comparable to those observed in younger patients. Titration of pregabalin to the lowest effective dose should allow for effective pain relief while minimizing AEs in older patients with neuropathic pain. Given the common use of polypharmacy in older patients, the absence of known drug-drug interactions makes pregabalin an important treatment option for older patients with pain of neuropathic origin.

## Background

Chronic neuropathic pain conditions, such as painful diabetic peripheral neuropathy (DPN) and postherpetic neuralgia (PHN), can be challenging to manage in older patients. Older patients tend to have multiple medical conditions and take several medications, which complicate treatment decisions. Concerns include the potential for drug-drug and drug-disease interactions and age-related changes in drug absorption, metabolism, and excretion [1-4]. Several studies have shown that high proportions (e.g. 34%-50%) of older patients (age ≥65 years) with neuropathic pain conditions had evidence of potentially inappropriate pain medication use based on a contraindication, warning, or potential drug-drug interaction [5,6]. Propoxyphene, tertiary tricyclic antidepressants (TCAs) (e.g. amitriptyline), and benzodiazepines were the most commonly used inappropriate medications in one study of elderly patients with neuropathic pain [5]. In patients with cardiovascular comorbidities, TCAs have been associated with a dose-related increased risk of sudden cardiac death and thus should be used with caution [7].

Pregabalin is a calcium channel α_2_δ ligand with analgesic, anxiolytic, and anticonvulsant properties that is minimally metabolized with renal excretion, displays linear gastrointestinal absorption leading to a predictable dose-response relationship, and has no known drug-drug interactions [8]. Clinical trials have shown the efficacy and safety of pregabalin at dosages ranging from 150 to 600 mg/day in patients ≥18 years of age with painful DPN or PHN [9-17]. In these studies, patients with DPN or PHN who received pregabalin at dosages of 300 mg/day [9,10,12,13,15] or 600 mg/day [9-11,14,15,17] experienced significant reductions in pain compared with placebo. Pregabalin administered at flexible dosages of 150 to 600 mg/day depending on patient response and tolerability was also shown to reduce endpoint mean pain compared with placebo in patients with DPN or PHN [14]. Across studies, results were mixed for the 150-mg/day dosage, with significant pain reductions observed in 2 studies of patients with PHN [13,15] but not in another study of patients with DPN [11]. Pregabalin 75 mg/day did not significantly reduce pain in DPN [10]. One study of patients with DPN showed significant differences on endpoint mean pain only in the group that received 600 mg/day, but not in the groups who received 150 or 300 mg/day [16].

Clinical studies of therapies for chronic pain typically lack sufficient numbers of older patients to evaluate the safety and efficacy of analgesics in this population. Current evidence suggests that selection of treatment for neuropathic pain in older patients should be similar to that for younger patients, with the exception of slower, more cautious dosing and consideration for issues unique to the older population [1]. The objective of this post hoc analysis was to evaluate the efficacy and safety of pregabalin in older patients with neuropathic pain using pooled double-blind, randomized, placebo-controlled studies of pregabalin in patients with DPN or PHN.

## Methods

### Clinical Study Selection

The goal of this analysis was to break down efficacy and safety data by specific age cut-offs, which would have been difficult or impossible to obtain using summary statistics from published reports. Furthermore, it has been suggested that study-level analyses can lead to biased assessments, and use of aggregated summary values has some limitations for explaining the heterogeneity of results [18-20]. Access to a rich, in-house, patient-level database provided us with the flexibility to analyze data using specific age cut-offs and allowed for increased precision of our estimates. Thus, this post hoc analysis was based entirely on data from in-house, Pfizer Inc.-sponsored, clinical studies. Clinical studies of patients with DPN or PHN were pooled if they met the following criteria: 1) were Pfizer Inc.-sponsored studies completed prior to the end of 2006; 2) were randomized, parallel, placebo-controlled, and double-blind; 3) had at least 1 fixed-dose pregabalin treatment arm; 4) had in-house, patient-level efficacy and safety data available; 5) had similar treatment durations; and 6) had same primary outcome. Of the 22 Pfizer Inc.-funded clinical studies of patients with DPN and/or PHN that were completed prior to the end of 2006, 8 studies were excluded from this analysis because they failed to meet criterion 2 (e.g. open-label); 2 studies did not meet criterion 3 (e.g.; only flexible-dose pregabalin arms) and 1 study did not meet criterion 6.

### Post hoc Analysis

Data from 11 double-blind, randomized, placebo-controlled, Pfizer Inc.-sponsored studies that evaluated the efficacy and safety of pregabalin in patients with DPN or PHN were used for this analysis. Results from 9 of these studies have been reported [9-17]. Results of the other 2 studies, 1008-030 and 1008-040, have been summarized in a review article [21] and a European Public Assessment Report Scientific Discussion posted at the European Medicines Agency Web site [22]; additionally, a synopsis of study 1008-040 has been posted at the PhRMA Clinical Study Results Web site [23]. The studies that met the selection criteria had a similar design with a 1-week baseline period followed by 5 to 13 weeks of placebo-controlled, double-blind treatment. Most included a 1-week titration period in the double-blind phase; 1 study had no titration period [16] and another had a 2-week titration period [11]. In these studies, patients were randomized to receive pregabalin at fixed dosages ranging from 75 to 600 mg/day or placebo. One study included a flexible-dose pregabalin 150- to 600-mg/day treatment arm [14].

All studies were conducted in compliance with the Declaration of Helsinki and the International Conference on Harmonization Good Clinical Practice Guideline. The final study protocols, any amendments, and informed consent documentation were approved by the Institutional Review Board(s) and/or Independent Ethics Committee(s) at each investigational center. The clinical protocols were conducted in accordance with Food and Drug Administration Regulations.

### Patient Population

Eligible patients were male and female patients aged ≥18 years with DPN or PHN; one study required patients at sites in Austria to be ≥19 years of age. Patients with DPN had a diagnosis of diabetes mellitus type 1 or type 2 and a diagnosis of painful DPN for ≥1 years, except for 2 studies that included patients with painful DPN for ≥3 months [17] and ≥6 months [14]. Patients with PHN must have had pain present for ≥3 months [9,14,15] or >6 months [13] after healing of herpes zoster rash, depending on the study. Female patients were required to be nonpregnant, not lactating, surgically sterile, postmenopausal, or use an effective form of contraception. Patients were included if they had scores ≥40 mm on the visual analog scale of the Short-Form McGill Pain Questionnaire at screening and randomization, and had an average Daily Pain Rating Scale (DPRS) score ≥4 derived from at least 4 diary entries during the 1-week baseline period. Patients were excluded if they had low creatinine clearance (CL_cr_; defined as ≤30 mL/min [9,15,16] or ≤60 mL/min [10,12,14,17] depending on the study). In 2 studies of patients with PHN [9,15], the dose of pregabalin was adjusted based on baseline renal function. Patients with CL_cr _>60 mL/min received pregabalin 600 mg/day and those with CL_cr _values of >30 to 60 mL/min were assigned to 300 mg/day. Patients with DPN were required to have hemoglobin A_1c _levels ≤11%. Six of the 11 studies excluded patients who had previously failed to respond to gabapentin at dosages ≥1200 mg/day for the treatment of DPN or PHN [9,10,12,13,22,23].

### Efficacy and Safety Assessments

The primary efficacy measure was the endpoint average pain score on DPRS (0 = no pain to 10 = worst possible pain). Endpoint mean pain was based on patients' pain score ratings over the last 7 days of treatment. The Initiative on Methods, Measurement, and Pain Assessment in Clinical Trials (IMMPACT) group recommends the use of responder analyses rather than mean changes in pain to determine clinically meaningful change in pain in clinical trials of chronic pain [24]. Patients who experience a ≥30% reduction in pain are considered to have a moderately important improvement in pain and those who experience a ≥50% reduction in pain, a substantial improvement in pain [24]. Furthermore, a pain score of ≤3 on the DPRS (no worse than mild pain) at endpoint represents an ideal outcome for patients with chronic pain [25]. Other outcome measures included sleep interference score on Daily Sleep Interference Scale (DSIS; 0 = pain did not interfere with sleep to 10 = pain completely interfered with sleep) and safety. Both pain and sleep interference scores on the DPRS and DSIS, respectively, were recorded daily by patients in diaries upon waking. Safety was assessed from treatment-emergent adverse events (AEs) and study discontinuations owing to AEs reported during the studies.

### Statistical Analyses

For the pooled analysis, patients with DPN or PHN were combined and stratified into the following age groups: 18 to 64 years, 65 to 74 years, and ≥75 years. Only data for pregabalin at dosages of 150 to 600 mg/day were evaluated because 75 mg/day is not an approved dosage. Demographics and baseline characteristics (mean DPRS and DSIS sleep interference scores) were summarized descriptively by treatment and disease groups.

To explore the differential effects on pain among age groups, a treatment-by-age interaction analysis was performed on patients using analysis of covariance with terms for treatment, age category, protocol, baseline pain, and treatment-by-age category interaction. Last observation carried forward (LOCF) was used to impute missing values for the evaluation of response rates for endpoint pain score ≤3 and 30% and 50% pain relief. The more conservative baseline observation carried forward (BOCF) method of imputation was also used to evaluate response rates for both 30% and 50% pain relief, but not endpoint pain score ≤3 since patients were required to have a pain score ≥4 on the DPRS at baseline for inclusion in these studies. The most common AEs (≥10% of any age or treatment group) were tabulated by treatment and age groups. The 95% confidence intervals (CI) for relative risks for pregabalin 300 mg/day (most common dosage) versus placebo were displayed by age groups.

## Results

**Figure 1:**
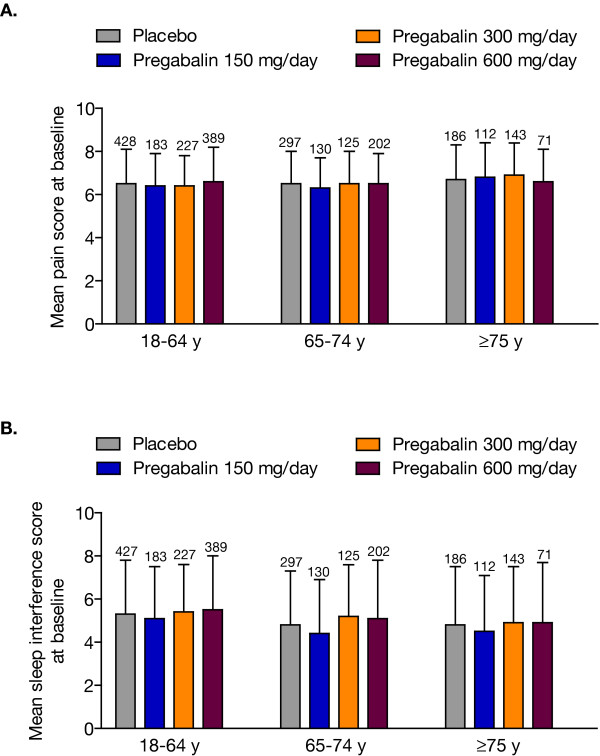
**Baseline mean pain (A) and sleep interference (B) scores by age and dosage group**. Error bars represent standard deviation of the mean. Number above each bar is number of patients.

In total, 2516 patients were included in the pooled analysis: 1513 patients with DPN and 1003 patients with PHN. In the overall pooled group, 93.2% were white, 53.5% were male, and 50.9% were ≥65 years of age. Among patients with DPN, 57.6% were male and 32.6% were ≥65 years of age; among patients with PHN, 47.5% were male and 78.4% were ≥65 years of age (Table 1). Baseline mean pain scores and mean sleep interference scores were comparable among age and dosage groups (Figure 1).

**Table 1:** Demographic and clinical characteristics at baseline

Characteristic	Diabetic peripheral neuropathy				Postherpetic neuralgia			
		
	Placebo (n = 558)	Pregabalin 150 mg/day (n = 176)	Pregabalin 300 mg/day (n = 266)	Pregabalin 600 mg/day (n = 513)	Placebo (n = 363)	Pregabalin 150 mg/day (n = 251)	Pregabalin 300 mg/day (n = 230)	Pregabalin 600 mg/day (n = 159)
Male, n (%)	308 (55.2)	110 (62.5)	144 (54.1)	309 (60.2)	180 (49.6)	117 (46.6)	103 (44.8)	76 (47.8)
Age, n (%)								
18-64 y	366 (65.6)	127 (72.2)	182 (68.4)	344 (67.1)	68 (18.7)	57 (22.7)	45 (19.6)	47 (29.5)
65-74 y	158 (28.3)	39 (22.2)	62 (23.3)	139 (27.1)	143 (39.4)	92 (36.6)	64 (27.8)	69 (43.4)
≥75 y	34 (6.1)	10 (5.7)	22 (8.3)	30 (5.8)	152 (41.9)	102 (40.6)	121 (52.6)	43 (27.0)
Race								
White	496 (88.9)	167 (94.9)	243 (91.4)	460 (89.7)	354 (97.5)	244 (97.6)	226 (99.1)	154 (96.9)
Black	28 (5.0)	3 (1.7)	10 (3.8)	22 (4.3)	2 (0.5)	4 (1.6)	2 (0.9)	1 (0.6)
Hispanic	24 (4.3)	2 (1.1)	7 (2.6)	20 (3.9)	4 (1.1)	2 (0.8)	1 (0.4)	4 (2.5)
Asian or Pacific Islander	2 (0.4)	2 (1.1)	3 (1.1)	5 (1.0)	2 (0.5)	1 (0.4)	0	0
Alaskan or Native American	1 (0.2)	0	1 (0.4)	0	0	0	0	0
Other	7 (1.3)	2 (1.1)	2 (0.8)	6 (1.2)	1 (0.3)	0	1 (0.4)	0
Weight, kg, mean (SD)	92.6 (20.6)	91.1 (17.8)	93.6 (20.1)	93.1 (19.8)	74.7 (15.7)	73.9 (15.6)	69.8 (13.6)	77.8 (13.9)
Height, cm, mean (SD)	170.8 (10.1)	172.3 (10.2)	171.4 (10.1)	171.2 (9.9)	167.0 (9.9)	165.5 (9.8)	165.3 (10.5)	166.3 (11.1)
CL_cr_, mL/min								
Mean (SD)	101.9 (37.3)	101.0 (33.0)	101.7 (36.7)	98.5 (30.3)	69.9 (26.5)	68.7 (24.3)	63.2 (24.3)	84.8 (22.3)
N	554	175	265	511	362	250	230	156
Baseline pain^a^								
Mean (SD)	6.5 (1.6)	6.3 (1.4)	6.4 (1.4)	6.6 (1.6)	6.6 (1.5)	6.6 (1.6)	6.8 (1.5)	6.5 (1.5)
N	550	175	265	508	361	250	230	154
Baseline sleep^b^								
Mean (SD)	5.3 (2.4)	5.1 (2.4)	5.6 (2.2)	5.4 (2.6)	4.6 (2.7)	4.5 (2.5)	4.8 (2.6)	4.8 (2.4)
N	549	175	265	508	361	250	230	154

^a ^Daily Pain Rating Scale score

^b ^Daily Sleep Interference Scale score

SD: standard deviation; CL_cr_: creatinine clearance

**Figure 2:**
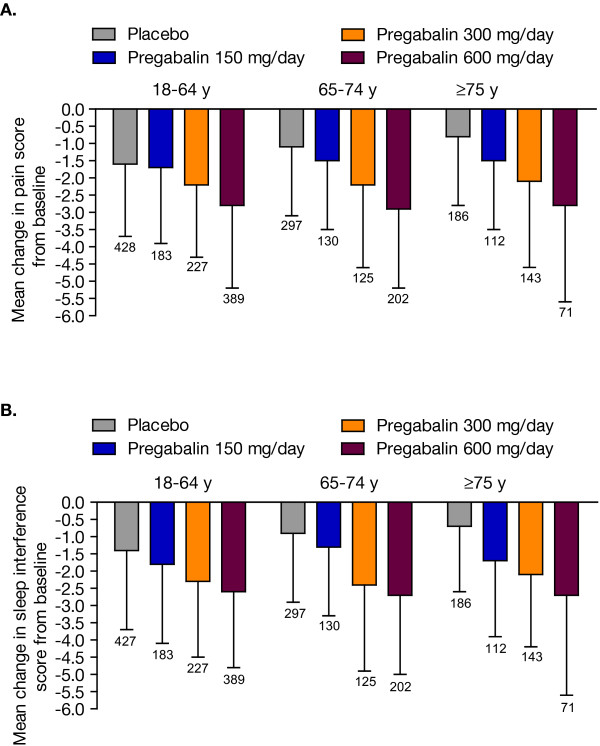
**Change in mean pain (A) and sleep interference (B) scores at endpoint**. Error bars represent standard deviation of the mean. Number above each bar is number of patients.

Comparable dose-related improvements in endpoint mean pain score were observed for pregabalin across age groups (Figure 2A). Similar results were observed for improvements in endpoint mean sleep interference scores (Figure 2B). Interestingly, the placebo response on pain and sleep interference scores appeared higher in the younger age group compared with the older age groups. While these results suggested a trend for increasing pregabalin-mediated pain reductions with increasing age, driven by an inverse relationship between placebo response and age, it was not statistically significant. Placebo-corrected least squares mean differences in pain with pregabalin between age groups were -0.155 (95% CI: -0.412, 0.109; p = 0.2497) for patients aged 18 to 64 years versus ≥75 years; -0.157 (95% CI: -0.419, 0.105; p = 0.2402) for patients aged 65 to 74 years versus ≥75 years; and 0.002 (95% CI: -0.215, 0.218; p = 0.9882) for patients aged 18 to 64 years versus 65 to 74 years.

To better understand the apparent differences in placebo response among age groups, we defined a contrast in our model in the placebo group. Overall, there were significant differences among age groups in placebo patients with respect to pain relief (p = 0.005), indicating a trend for decreasing placebo response with older age. Placebo patients aged 18 to 64 years showed the largest improvement in average pain score (-1.47) compared with either placebo patients aged 65 to 74 years (-1.05; p = 0.0112) or aged ≥75 years (-0.86; p = 0.0031). No significant differences in placebo pain response were observed between those aged 65 to 74 years and those aged ≥75 years (p = 0.3318).

**Figure 3:**
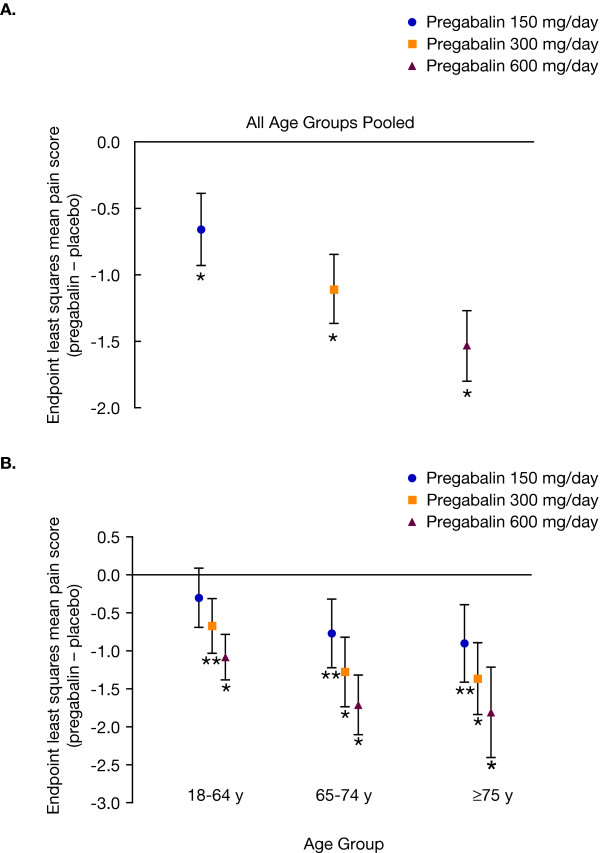
**Placebo-adjusted endpoint mean pain by pregabalin dosage**. The upper panel (A) shows placebo-adjusted endpoint mean pain for pooled age groups. The lower panel (B) shows placebo-adjusted endpoint mean pain by age and dosage group. Bars represent the 95% confidence interval. *p < 0.0001 versus placebo. **p ≤ 0.0009 versus placebo.

Significant dose-dependent reductions in endpoint mean pain score on DPRS were observed for pregabalin dosages of 150, 300, and 600 mg/day versus placebo for pooled age groups (p < 0.0001; Figure 3A). For patients aged ≥75 years, significant improvements in endpoint mean pain score were observed for pregabalin versus placebo at all dosages (150 mg/day pregabalin-placebo difference, -0.90 [p = 0.0005]; 300 mg/day pregabalin-placebo difference, -1.37 [p < 0.0001]; 600 mg/day pregabalin-placebo difference, -1.81 [p < 0.0001]; Figure 3B). Significant differences in placebo-corrected endpoint mean pain were also observed for all pregabalin dosages in patients aged 65 to 74 years (150 mg/day pregabalin-placebo difference, -0.77 [p = 0.0009]; 300 mg/day pregabalin-placebo difference, -1.28 [p < 0.0001]; 600 mg/day pregabalin-placebo difference, -1.71 [p < 0.0001]; Figure 3B). In patients aged 18 to 65 years, pregabalin provided significant improvements in the 300-mg/day (pregabalin-placebo difference, -0.67 [p = 0.0003]) and 600-mg/day (pregabalin-placebo difference, -1.08 [p < 0.0001]) dosage groups, but not the 150-mg/day group (pregabalin-placebo difference, -0.30 [p = 0.128]; Figure 3B).

Because of the heterogeneity of response in patients with neuropathic pain, it is important not only to look at mean changes in pain, but also the proportion of patients with clinically defined response criteria. Generally, higher response rates were observed for ≥30% pain relief, ≥50% pain relief, and pain score at endpoint ≤3 with increasing pregabalin dose in all age groups (Table 2). Moderately important improvements in pain (≥30% reduction) were observed in one-third to more than one-half of patients and substantial improvements in pain (≥50% reduction) in one-fifth to nearly one-half of patients who received 150 to 600 mg/day pregabalin across age groups regardless of the method of imputation (Table 2). Furthermore, one-quarter to nearly one-half of patients had pain scores ≤3 at endpoint reflecting mild pain following treatment with 150 to 600 mg/day pregabalin (Table 2).

**Table 2:** Clinically important pain outcomes by age and dose group

	≥30% Pain relief, n (%)		≥50% Pain relief, n (%)		Endpoint pain score ≤3, n (%)*
			
	BOCF	LOCF	BOCF	LOCF	
18-64 years					
Placebo	153 (35.7)	163 (38.1)	94 (22.0)	98 (22.9)	111 (25.9)
Pregabalin 150 mg/d	72 (39.3)	74 (40.4)	45 (24.6)	46 (25.1)	50 (27.3)
Pregabalin 300 mg/d	109 (48.0)	120 (52.9)	73 (32.2)	82 (36.1)	92 (40.5)
Pregabalin 600 mg/d	199 (51.3)	236 (60.8)	153 (39.4)	179 (46.1)	170 (43.8)
65-74 years					
Placebo	73 (24.6)	80 (26.9)	47 (15.8)	51 (17.2)	57 (19.2)
Pregabalin 150 mg/d	45 (34.6)	48 (36.9)	31 (23.8)	32 (24.6)	34 (26.2)
Pregabalin 300 mg/d	56 (44.8)	68 (54.4)	38 (30.4)	46 (36.8)	43 (34.4)
Pregabalin 600 mg/d	109 (54.0)	132 (65.3)	83 (41.1)	99 (49.0)	91 (45.0)
≥75 years					
Placebo	39 (21.0)	42 (22.6)	22 (11.8)	23 (12.4)	24 (12.9)
Pregabalin 150 mg/d	40 (35.7)	46 (41.1)	28 (25.0)	32 (28.6)	36 (32.1)
Pregabalin 300 mg/d	43 (30.1)	64 (44.8)	30 (21.0)	42 (29.4)	38 (26.6)
Pregabalin 600 mg/d	24 (33.8)	41 (57.7)	18 (25.4)	32 (45.1)	35 (49.3)

LOCF, last observation carried forward; BOCF, baseline observation carried forward.

*Pain score ≤3 at endpoint on the Daily Pain Rating Scale (0 = no pain to 10 = worst possible pain) based on LOCF analysis.

**Figure 4:**
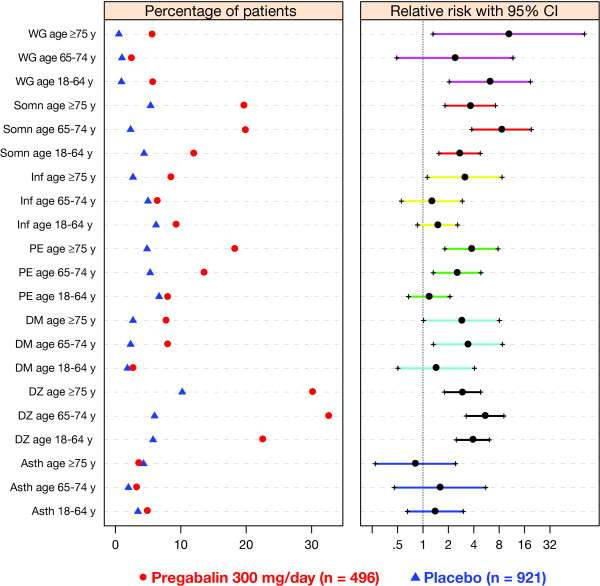
**Relative risks for the most frequent adverse events for pregabalin 300 mg/day versus placebo by age group**. The left panel shows the percentage of patients in each age group that reported a particular adverse event by treatment group. The right panel shows the relative risk of given adverse events in each age group for pregabalin versus placebo. CI: confidence interval; WG: weight gain; Somn: somnolence; Inf: infection; PE: peripheral edema; DM: dry mouth; DZ: dizziness; Asth: asthenia.

The most common AEs that occurred in ≥10% of any age or treatment group were dizziness, somnolence, peripheral edema, asthenia, dry mouth, weight gain, and infection. In patients with either DPN or PHN, the percentage of patients with a given AE was not noticeably different in patients aged ≥75 years versus those aged 65 to 74 years (Table 3). The relative risks for the most common AEs for pregabalin versus placebo are shown for pregabalin 300 mg/day, a dosage commonly used for the treatment of neuropathic pain (Figure 4). While an increased risk ( > 1) was observed for several AEs relative placebo (e.g. somnolence and dizziness), the point estimates for the relative risks did not uniformly increase with older age and the corresponding 95% CI overlapped among age groups (Figure 4). The relative risks for the most common AEs increased with pregabalin dose, but appeared unrelated to age regardless of dose (Figure 4 and Additional files 1 and 2). Study discontinuations owing to AEs were generally higher for pregabalin 300 mg/day and 600 mg/day compared with placebo or pregabalin 150 mg/day across all age groups (Table 4). A trend for higher AE-related discontinuations with increasing age was observed, particularly in patients with PHN at the higher pregabalin doses.

**Table 3:** Most common adverse events by treatment group, age, and type of neuropathic pain

	Placebo, n (%)^a^		Pregabalin 150 mg/day, n (%)^a^		Pregabalin 300 mg/day, n (%)^a^		Pregabalin 600 mg/day, n (%)^a^	
				
Adverse event	DPN (n = 558)	PHN (n = 363)	DPN (n = 176)	PHN (n = 251)	DPN (n = 266)	PHN (n = 230)	DPN (n = 513)	PHN (n = 159)
Dizziness								
Age 18-64 y	16 (4.4)	9 (13.2)	7 (5.5)	4 (7.0)	40 (22.0)	11 (24.0)	85 (24.7)	23 (48.9)
Age 65-74 y	8 (5.1)	10 (7.0)	3 (7.7)	13 (14.1)	16 (25.8)	25 (39.1)	46 (33.1)	25 (36.2)
Age ≥75 y	2 (5.9)	17 (11.2)	2 (20.0)	22 (21.6)	6 (27.3)	37 (30.6)	11 (36.7)	13 (30.2)
Somnolence								
Age 18-64 y	14 (3.8)	5 (7.4)	5 (3.9)	7 (12.3)	24 (13.2)	3 (6.7)	45 (13.1)	13 (27.7)
Age 65-74 y	2 (1.3)	5 (3.5)	2 (5.1)	9 (9.8)	11 (17.7)	14 (21.9)	16 (11.5)	20 (29.0)
Age ≥75 y	0	10 (6.6)	2 (20.0)	12 (11.8)	3 (13.6)	25 (20.7)	7 (23.3)	11 (25.6)
Peripheral edema								
Age 18-64 y	27 (7.4)	2 (2.9)	6 (4.7)	3 (5.3)	15 (8.2)	3 (6.7)	53 (15.4)	6 (12.8)
Age 65-74 y	10 (6.3)	6 (4.2)	3 (7.7)	9 (9.8)	9 (14.5)	8 (12.5)	24 (17.3)	12 (17.4)
Age ≥75 y	3 (8.8)	6 (3.9)	1 (10.0)	7 (6.9)	2 (9.1)	24 (19.8)	5 (16.7)	4 (9.3)
Asthenia								
Age 18-64 y	11 (3.0)	4 (5.9)	3 (2.4)	2 (3.5)	8 (4.4)	3 (6.7)	24 (7.0)	3 (6.4)
Age 65-74 y	0	6 (4.2)	0	6 (6.5)	4 (6.5)	0	14 (10.1)	7 (10.1)
Age ≥75 y	1 (2.9)	7 (4.6)	1 (10.0)	5 (4.9)	1 (4.5)	4 (3.3)	6 (20.0)	2 (4.7)
Dry mouth								
Age 18-64 y	6 (1.6)	2 (2.9)	1 (0.8)	5 (8.8)	6 (3.3)	0	18 (5.2)	8 (17.0)
Age 65-74 y	1 (0.6)	6 (4.2)	0	9 (9.8)	4 (6.5)	6 (9.4)	10 (7.2)	9 (13.0)
Age ≥75 y	0	5 (3.3)	2 (20.0)	5 (4.9)	3 (13.6)	8 (6.6)	2 (6.7)	6 (14.0)
Weight gain								
Age 18-64 y	3 (0.8)	1 (1.5)	6 (4.7)	3 (5.3)	9 (4.9)	4 (8.9)	34 (9.9)	5 (10.6)
Age 65-74 y	1 (0.6)	2 (1.4)	1 (2.6)	1 (1.1)	1 (1.6)	2 (3.1)	10 (7.2)	9 (13.0)
Age ≥75 y	1 (2.9)	0	1 (10.0)	1 (1.0)	0	8 (6.6)	1 (3.3)	5 (11.6)
Infection								
Age 18-64 y	25 (6.8)	2 (2.9)	10 (7.9)	9 (15.8)	17 (9.3)	4 (8.9)	10 (2.9)	1 (2.1)
Age 65−74 y	8 (5.1)	7 (4.9)	4 (10.3)	7 (7.6)	5 (8.1)	3 (4.7)	6 (4.3)	1 (1.4)
Age ≥75 y	2 (5.9)	3 (2.0)	0	6 (5.9)	1 (4.5)	11 (9.1)	1 (3.3)	2 (4.7)

^a^See Table 1 for total number of patients in each age group by treatment group and neuropathic pain condition

DPN: diabetic peripheral neuropathy; PHN: postherpetic neuralgia

**Table 4:** Discontinuations owing to adverse events by age, treatment group, and neuropathic pain condition

	Placebo, n (%)^a^	Pregabalin 150 mg/day, n (%)^a^	Pregabalin 300 mg/day, n (%)^a^	Pregabalin 600 mg/day, n (%)^a^
DPN				
Age 18-64 y	14 (3.8)	3 (2.4)	14 (7.7)	42 (12.2)
Age 65-74 y	12 (7.6)	3 (7.7)	9 (14.5)	26 (18.7)
Age ≥75 y	3 (8.8)	1 (10.0)	3 (13.6)	7 (23.3)

PHN				
Age 18-64 y	4 (5.9)	5 (8.8)	3 (6.7)	12 (25.5)
Age 65-74 y	11 (7.7)	5 (5.4)	8 (12.5)	15 (21.7)
Age ≥75 y	9 (5.9)	11 (10.8)	32 (26.4)	13 (30.2)

^a^See Table 1 for total number of patients in each age group by treatment group and neuropathic pain condition

DPN: diabetic peripheral neuropathy; PHN: postherpetic neuralgia

## Discussion

The findings from this post hoc pooled analysis showed that the efficacy and safety of pregabalin in older patients with neuropathic pain are comparable to those observed in younger patients with neuropathic pain. Clinically meaningful and robust efficacy for pregabalin was observed in both older and younger patients, even using the more conservative BOCF method for imputing missing data. Additionally, the response rates in this study for ≥30% pain relief and ≥50% pain relief were within the range of those observed in a systematic review of pregabalin in patients with PHN and DPN [26]. In this very difficult to treat patient population, up to 49% of patients finished treatment with pregabalin at a low pain state of pain scores ≤3, which is commonly associated with significantly less functional impairment and improved activities of daily living compared with moderate and severe pain states [27]. The relative risks for the most common AEs with pregabalin did not appear to be compromised by increasing age or type of neuropathic pain; however, a trend between AE-related discontinuations and age in patients was observed, particularly in patients with PHN. A study of patients in general practice in the United Kingdom found that nearly half of all patients newly prescribed pregabalin for neuropathic pain were aged ≥65 years [28]. This underscores the importance of better understanding the efficacy and safety of pregabalin in older patients.

In all but one of the studies in this analysis, patients were assigned a dose of pregabalin (or placebo) based on the randomization schema of the study and not titrated based on response to pregabalin. Renal excretion is compromised in up to one-half of older patients [4], which potentially affects plasma levels of pregabalin. In some, but not all, studies in this pooled analysis, the assigned dosage of pregabalin (e.g. 600 mg/day) was reduced (e.g. 300 mg/day) based on the patient's renal function (Cl_cr_) at baseline. Because of these age-related impairments and varying criteria for dose reductions based on CL_cr _measurements, it cannot be assumed that patients in a particular dosage group had identical exposure to pregabalin, and this may have influenced the incidence of AEs in the higher dosage groups. Given that all 3 dosages of pregabalin were shown to significantly reduce pain in older patients with neuropathic pain, it is feasible to assume that some of the patients assigned to the higher dosage group may have achieved adequate pain relief with a lower dosage of pregabalin. Dizziness and somnolence are the most common AEs among older patients and potentially can be minimized by initiating pregabalin at low doses and slowly titrating to a dose at which the patient experiences pain relief, while taking into account any impairment in renal function. Titration of pregabalin to the lowest effective dose may be especially important for older patients with PHN.

The high placebo response in patients aged 18 to 64 years in the current pooled analysis may partially explain the lack of significant reduction in endpoint mean pain score for the pregabalin 150-mg/day dosage in this group. Interestingly, in the current analysis, a significant difference was found in placebo response on pain between younger (age <65 years) and older patients (age ≥65 years). A similar phenomenon has been observed in a pooled analysis of rizatriptan in patients with migraine [29]. It is unclear why the placebo response on mean pain scores differed between younger and older patients. Placebo response in clinical studies of pain may be driven by an expectation for benefit mediated via endogenous opioid and cholecystokinin pathway activation [30]. This might suggest that there is an effect of age on these systems. Perhaps younger patients with neuropathic pain enrolled in clinical studies have higher expectations for a benefit than older patients.

The choice of analgesic in patients with neuropathic pain of any age depends on the type of neuropathic pain, potential for AEs, comorbid conditions, and risk for drug-drug interactions. Comparison of the guidelines from the International Association for the Study of Pain Neuropathic Pain Special Interest Group, European Federation for Neurological Societies, and Canadian Pain Society found a consensus for the use of TCAs, gabapentin, and pregabalin as first-line treatments for neuropathic pain [31]. Older patients tend to take several medications and have concurrent medical conditions. The American Geriatric Society (AGS) recommends against the use of tertiary TCAs (e.g. amitriptyline) for the treatment of pain in older patients (age ≥75 years) because of safety risks including cardiovascular effects, orthostatic hypotension, and cognitive impairment [32]. Despite these AGS recommendations, several claims-based studies have found high rates of potentially inappropriate use of TCAs, in particular amitriptyline, in older patients with neuropathic pain conditions [5,33]. Analgesics metabolized by cytochrome P450 (e.g. duloxetine) should be used with caution in patients who take multiple medications because of the potential for drug-drug interactions with drugs that inhibit or are metabolized by cytochrome P450 [34]. Pregabalin is not metabolized by cytochrome P450 and has no known drug-drug interactions [8]; however, appropriate dose reductions should be made in older patients with renal impairment [34].

Whether the results from controlled clinical trials, which have strict patient enrollment criteria, are generalizable to patients encountered in clinical practice is always a concern. Pregabalin significantly reduced pain and sleep interference scores compared with pretreatment levels across a broad range of patients with refractory neuropathic pain in an open-label routine care setting [35]. Several real-world, retrospective, claims-based studies have confirmed the results of randomized controlled clinical studies showing a benefit for pregabalin in various neuropathic pain conditions. In patients with PHN, opioid use significantly decreased following initiation of pregabalin, whereas it increased following initiation of gabapentin [36]. In older patients (age ≥65 years) with fibromyalgia, many of whom had comorbid neuropathic pain conditions, initiation of pregabalin was associated with significantly fewer physician office visits and total outpatient visits compared with pretreatment levels [37]. Finally, in a general practice setting in the United Kingdom, use of pregabalin was associated with decreased use of other analgesics in patients with neuropathic pain conditions [28].

Limitations of this analysis include that it was a post hoc analysis. Unlike randomized clinical trials, the age and dosage groups were not balanced for the number of patients resulting in fewer patients evaluated in the older groups, especially at the highest pregabalin dosage. Analysis of data across multiple clinical trials with slightly different eligibility criteria and 2 different neuropathic pain conditions may have contributed to some of the variability in the results. Furthermore, large placebo responses in clinical trials of neuropathic pain may confound assessment of treatment differences. Because the study populations in this analysis were selected based on inclusion and exclusion criteria to express particular characteristics, the results may not be immediately generalizable to the patient population encountered in clinical practice.

## Conclusions

In this post hoc pooled analysis, pregabalin 150 to 600 mg/day reduced pain and improved sleep interference in older patients (age ≥65 years) with neuropathic pain. The improvements in pain in older patients were comparable to those observed in younger patients and clinically meaningful improvements in pain were observed in all age groups. The most common AEs were somnolence, weight gain, dry mouth, asthenia, dizziness, peripheral edema, and infection. The incidence of AEs did not appear to be related to older age or type of neuropathic pain, but did appear to be related to pregabalin dose. Slow titration of pregabalin to the lowest effective dose that provides pain relief should minimize the risk of AEs in older patients with neuropathic pain.

## List of Abbreviations Used

AEs: adverse events; AGS: American Geriatrics Society; CI: confidence interval; CL_cr_: creatinine clearance; DPN: diabetic peripheral neuropathy; DPRS: Daily Pain Rating Scale; DSIS: Daily Sleep Interference Scale; PHN: postherpetic neuralgia; TCAs: tricyclic antidepressants

## Competing interests

David Semel, T. Kevin Murphy, Gergana Zlateva, Raymond Cheung, and Birol Emir are full-time employees of Pfizer Inc.

## Authors' contributions

DS and GZ conceived the post hoc analysis and DS, GZ, TKM, RC, and BE participated in data analysis and interpretation. BE performed the statistical analyses. All authors participated in manuscript preparation and read and approved the final manuscript.

## Pre-publication history

The pre-publication history for this paper can be accessed here:

http://www.biomedcentral.com/1471-2296/11/85/prepub

## Supplementary Material

Additional file 1 **Relative risks for the most frequent adverse events for pregabalin 150 mg/day versus placebo by age group**. The left panel shows the percentage of patients in each age group that reported a particular adverse event by treatment group. The right panel shows the relative risk of given adverse events in each age group for pregabalin versus placebo. Click here for file

Additional file 2 **Relative risks for the most frequent adverse events for pregabalin 600 mg/day versus placebo by age group**. The left panel shows the percentage of patients in each age group that reported a particular adverse event by treatment group. The right panel shows the relative risk of given adverse events in each age group for pregabalin versus placebo. Click here for file
