# Supplementary material for: Evaluation of the safety and efficacy of pregabalin in older patients with neuropathic pain: results from a pooled analysis of 11 clinical studies
Source: BMC Fam Pract. 2010 Nov 5;11:85. doi: 10.1186/1471-2296-11-85 (PMC2988717; doi:10.1186/1471-2296-11-85)
Supplement: Additional file 1 — Relative risks for the most frequent adverse events for pregabalin 150 mg/day versus placebo by age group. The left panel shows the percentage of patients in each age group that reported a particular adverse event by treatment group. The right panel shows the relative risk of given adverse events in each age group for pregabalin versus placebo. [file 1471-2296-11-85-S1.PDF]

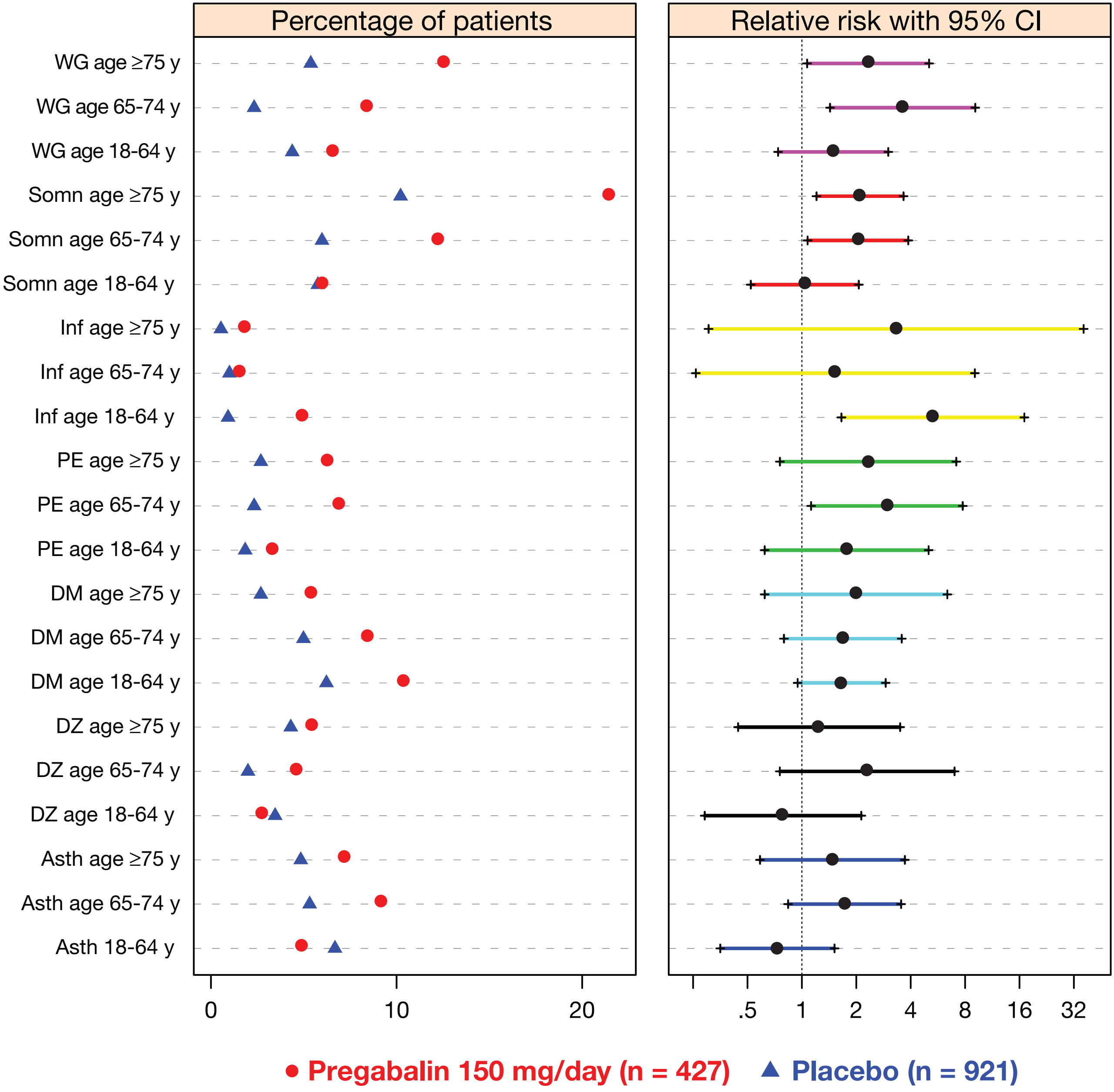

Additional file 1. Relative risks for the most frequent adverse events for pregabalin 150 mg/day versus placebo by age group. The left panel shows the percentage of patients in each age group that reported a particular adverse event by treatment group. The right panel shows the relative risk of given adverse events in each age group for pregabalin versus placebo. CI: confidence interval; WG: weight gain; Somn: somnolence; Inf: infection; PE: peripheral edema; DM: dry mouth; DZ: dizziness; Asth: asthenia.
